# Supplementary material for: Antimicrobial Peptide with a Bent Helix Motif Identified in Parasitic Flatworm Mesocestoides corti
Source: Int J Mol Sci. 2024 Oct 30;25(21):11690. doi: 10.3390/ijms252111690 (PMC11546468; doi:10.3390/ijms252111690)
Supplement: Supplementary file 1 [file ijms-25-11690-s001.zip › ijms-3278722-supplementary.pdf]

# Supplementary Materials:

# Antimicrobial peptide with a bent helix motif identified in parasitic flatworm *Mesocestoides corti*

Tomislav Rončević [1], Marco Gerdol [2], Sabrina Pacor [2], Ana Cvitanović [3], Anamarija Begić [4], Ivana Weber [4], Lucija Krce [4,] Andrea Caporale [5], Mario Mardirossian [2], Alessandro Tossi [2] and Larisa Zoranić [4,*]

[1] University of Split, Faculty of Science, Department of Biology, 21000 Split, Croatia

[2] University of Trieste, Department of Life Sciences, 34127 Trieste, Italy

[3] University of Zagreb, Faculty of Science, Department of Biology, 10000 Zagreb, Croatia

[4] University of Split, Faculty of Science, Department of Physics, 21000 Split, Croatia

[5] Institute of Crystallography, CNR, Basovizza, 34149 Trieste, Italy

***** Correspondence: larisaz@units.it ; Tel.: +385 21 619 221

## List of tables and figures

***Figure S1:*** *Expression profiles and multiple sequence alignment of mesco peptides.*
***Figure S2:*** *AFM images of untreated E. coli ATCC DH5α cells.*
***Table S1:*** *Results of bioinformatical analysis*
***Figure S3:*** *Snapshots and DSSP plots from simulations mesco-2 in water*
***Figure S4:*** *Snapshots of mesco-2- model1 simulations with membrane*
***Figure S5:*** *DSSP plots from mesco-2-model1 simulations with membrane*
***Figure S6:*** *Density profiles for mesco-2-model 1 simulations with membrane*
***Figure S7:*** *Distance analysis for mesco-2-model1 simulations with membrane*
***Figure S8:*** *Contacts analysis for mesco-2-model1 simulations with membrane*
***Figure S9:*** *Snapshots of mesco-2- model2 simulations with membrane*
***Figure S10:*** *DSSP plots from mesco-2-model2 simulations with membrane*
***Figure S11:*** *Density profiles for mesco-2-model2 simulations with membrane*
***Figure S12:*** *Distance analysis for mesco-2-model2 simulations with membrane*
***Figure S13:*** *Contacts analysis for mesco-2-model2 simulations with membrane*
***Figure S14:*** *Analytical RP-HPLC of mesco-2 peptide.*

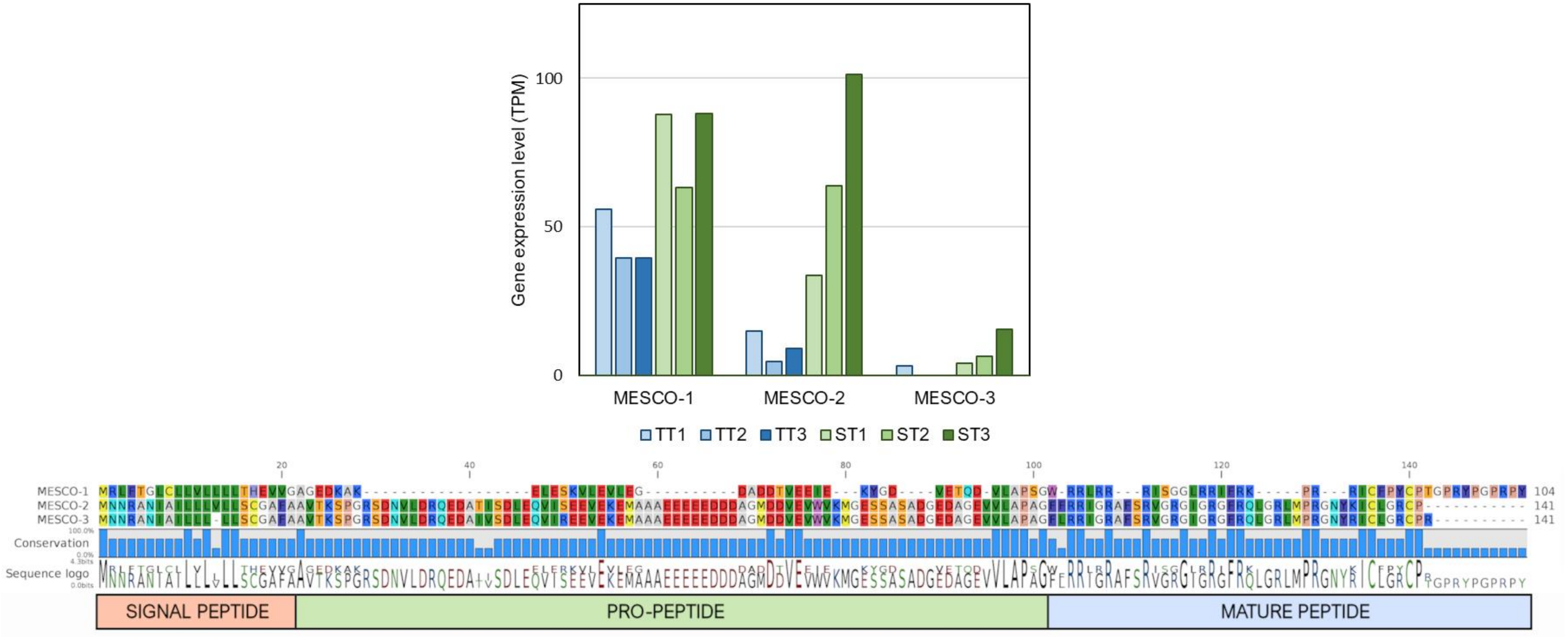


***Figure S1.*** The expression of the three complete genes was investigated in the larval (TT) and strobilated worm (ST) developmental stages, identifying a transcriptional level consistently higher in ST (upper image). Multiple sequence alignment of mesco peptides (lower image). The average sequence conservation is shown with a bar graph below the alignment and the consensus sequence of the mesco precursor peptide is shown as a sequence logo. The three main regions of the mesco peptides (signal peptide, mature region and C-terminal region) are indicated at the bottom part of the figure.

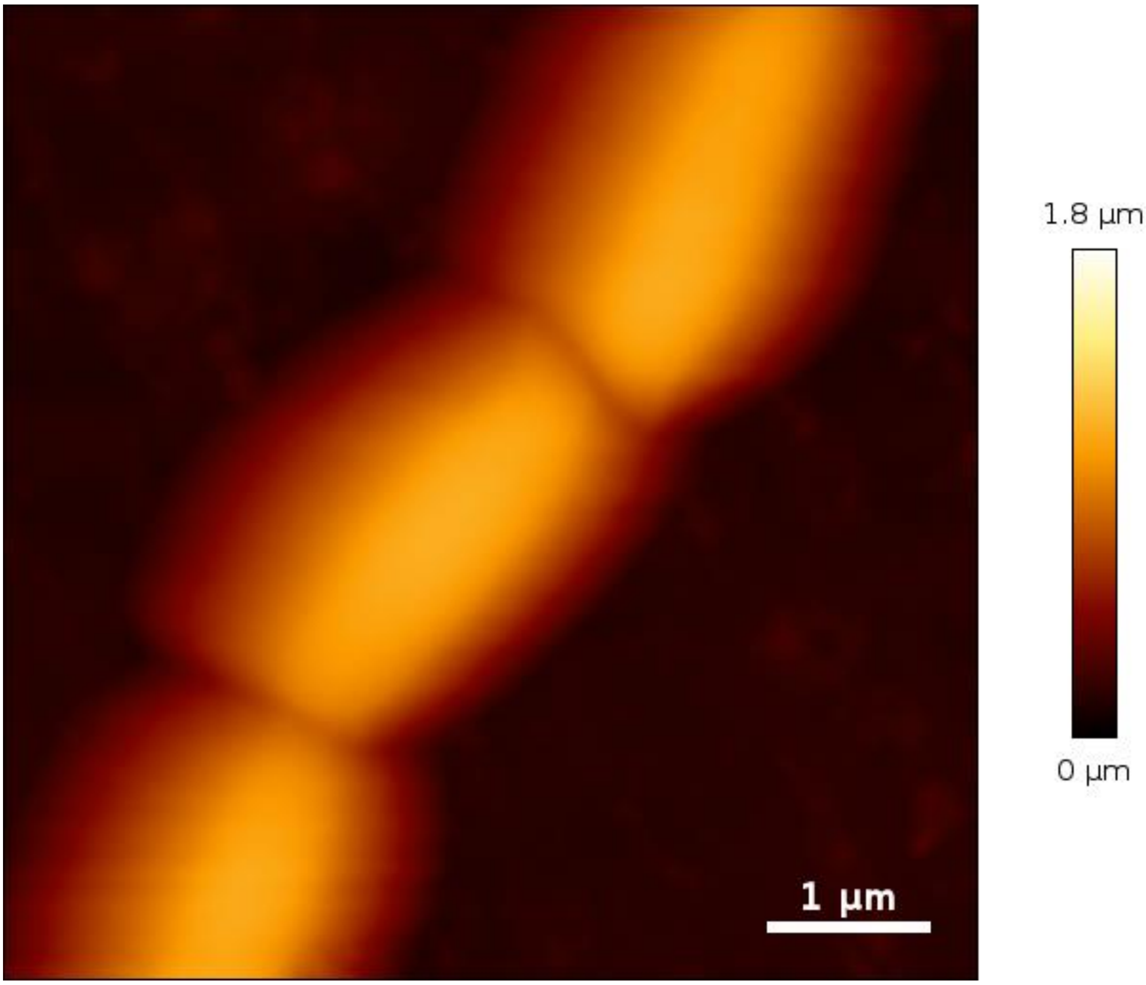


***Figure S2.*** AFM images of untreated E. coli ATCC DH5α cells. No damage was observed.

***Table S1*** Results of bioinformatical analysis. The CAMPR4 Support Vector Machine algorithm (accessed 2024 Jun 28) [1] and AmpGram (accessed 2024 Jun 28) servers were employed to calculate the antimicrobial peptide probabilities. Anticancer activity was classified using the ACPred server (accessed 2024 Jun 28) [2] and the mACPred server (accessed 2024 Jul 28). [3] was used to find the probability of anticancer activity. The PreAIP server (accessed 2024 Jun 28) [4] was used to predict anti-inflammatory activity, while antiviral activity was evaluated using ENNAVIA (accessed 2024 Jun 28) [5] and Meta-iAVP (accessed 2024 Jun 28) [6] servers. Antifungal prediction was conducted using the iAMPpred server (accessed 2024 Jun 29) [7]. The MLCPP server (accessed 2024 Jun 29) [8] was utilized to determine the probability of MESCO 2 being a cell-penetrating peptide. Toxicity prediction was performed with the ToxinPred server (accessed 2024 Jun 29) [9, 10].

| Feature | Server(s) Used | Probability/Score |
|---|---|---|
| **Cell-Penetrating** | MLCPP (https://balalab-skku.org/mlcpp2) | 0.848 |
| **Antimicrobial** | CAMPR$_4$ (http://www.camp.bicnirrh.res.in/predict) | 0.95-0.98 |
| | AmpGram (http://biongram.biotech.uni.wroc.pl/AmpGram) | 0.9094 |
| **Antibacterial** | iAMPpred (http://cabgrid.res.in:8080/amppred/server.php) | 0.98 |
| **Anticancer** | ACPred (http://codes.bio/acpred/) | 0.924 |
| | mACPred2 (https://balalab-skku.org/mACPpred2/) | 0.999 |
| **Antiviral** | ENNAVIA (https://research.timmons.eu/ennavia) | 1.0 |
| | iAMPpred (http://cabgrid.res.in:8080/amppred/server.php) | 0.93 |
| | Meta-iAVP (http://codes.bio/meta-iavp) | 0.744 |
| **Antifungal** | iAMPpred (http://cabgrid.res.in:8080/amppred/server.php) | 0.98 |
| **Anti-inflammatory** | PreAIP (http://kurata14.bio.kyutech.ac.jp/PreAIP/ ) | 0.596 (high confidence) |
| **Toxicity** | ToxinPred (https://webs.iiitd.edu.in/raghava/toxinpred/) | SVM score: -1.48<br>Non-toxin |

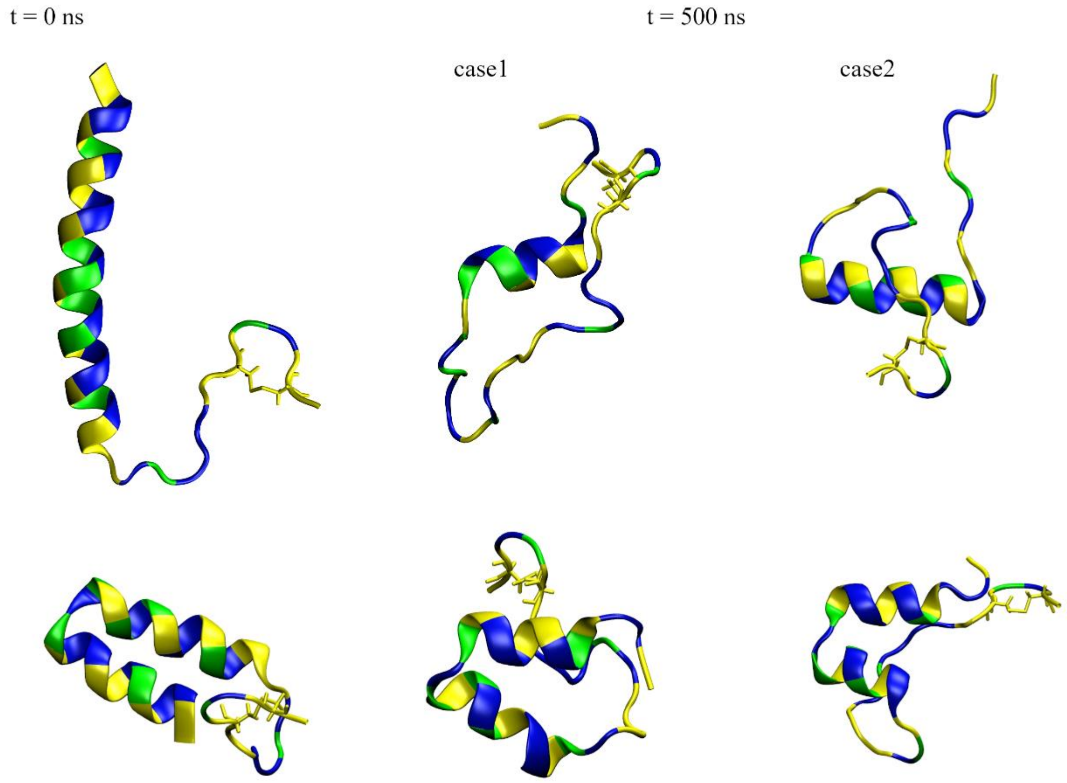


***Figure S3a*** Snapshots from the simulation of a single peptide in water, with the initial conformation depicted on the left and the one at 500 ns simulation time in the middle for case 1 and on the right for case2. The top row represents results for *model1*, while the bottom row to *model2* simulations. The peptides are shown in a ribbon representation, with polar residues colored in blue, hydrophobic residues in yellow, and glycine in green. Water molecules and ions are excluded for clarity.

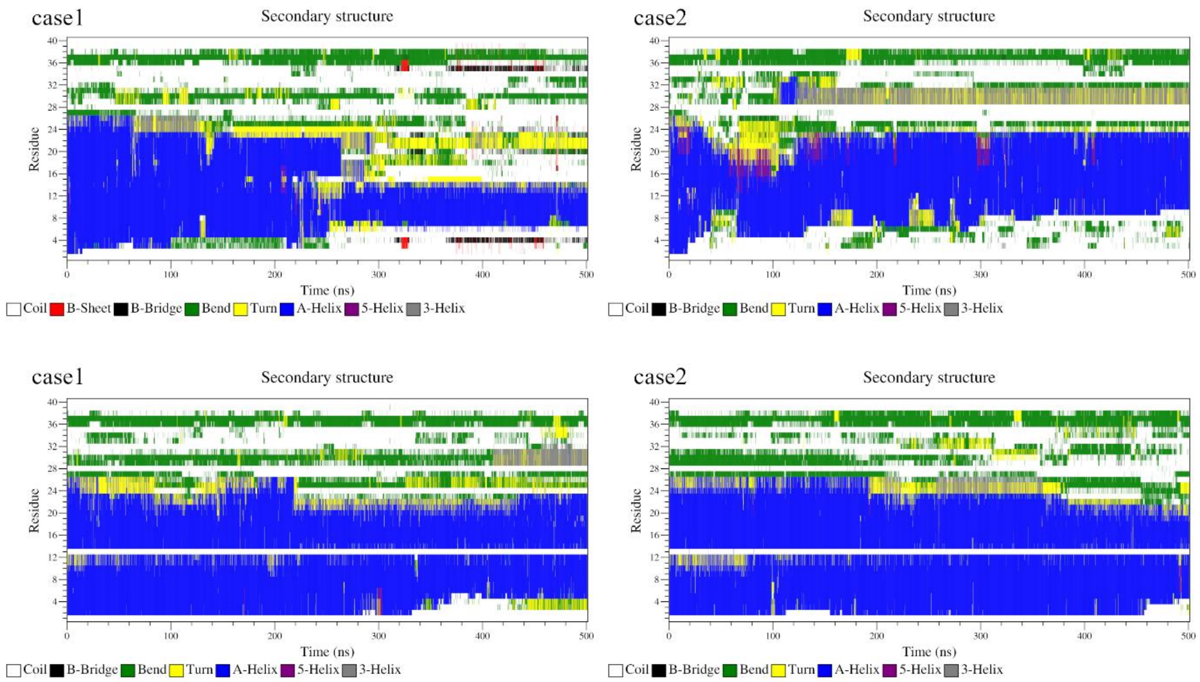


***Figure S3b*** Time evolution of secondary structures of mesco-2 *model1* (top) and *model2* (bottom) in water simulations case1 (left) and case2 (right), obtained by the DSSP [12] program.

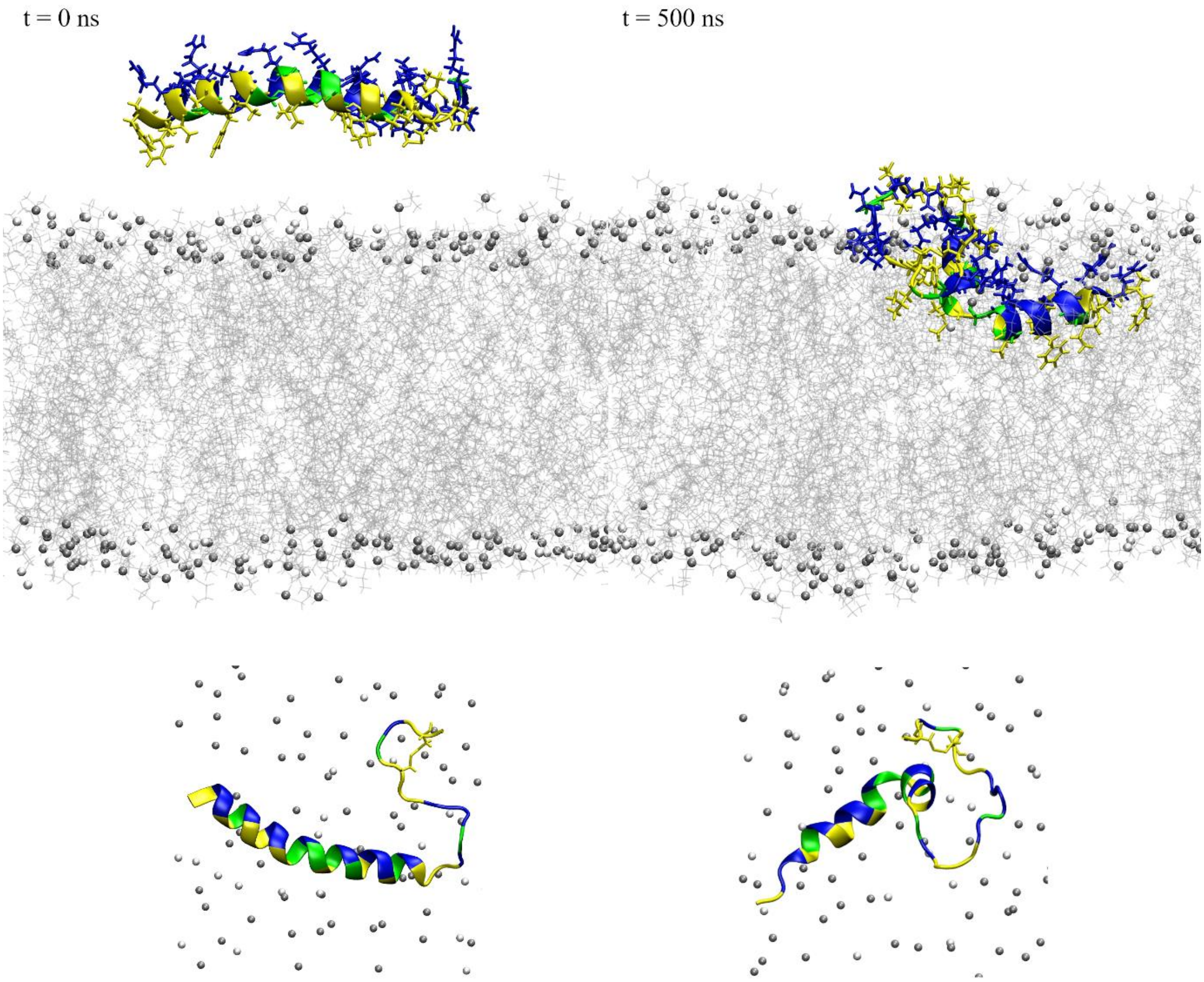


***Figure S4a*** Snapshots from the case1 simulation of a single peptide near POPE:POPG membrane for *model1,* with the initial conformation depicted on the left and the one at 500 ns simulation time on the right. The top row represents the side and bottom rows top view. The peptides are shown in a ribbon representation, with polar residues colored in blue, hydrophobic residues in yellow, and glycine in green. Lipids are represented in grey, with phosphorus atoms depicted as beads and acyl chains as lines. Water molecules and ions are excluded for clarity.

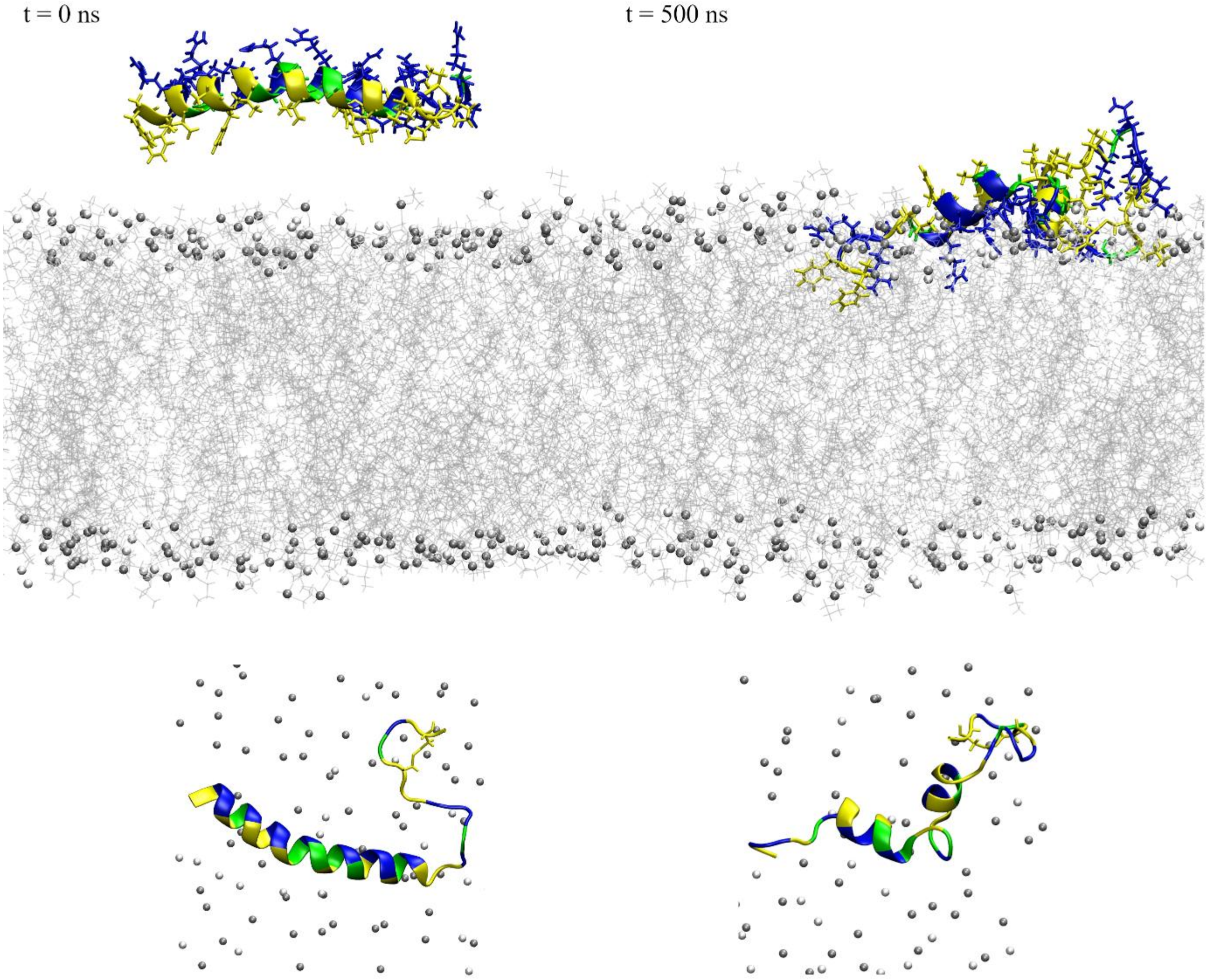


**Figure S4b** Snapshots from the case2 simulation of a single peptide near POPE:POPG membrane for *model1,* with the initial conformation depicted on the left and the one at 500 ns simulation time on the right. The top row represents the side and bottom rows top view. The peptides are shown in a ribbon representation, with polar residues colored in blue, hydrophobic residues in yellow, and glycine in green. Lipids are represented in grey, with phosphorus atoms depicted as beads and acyl chains as lines. Water molecules and ions are excluded for clarity.

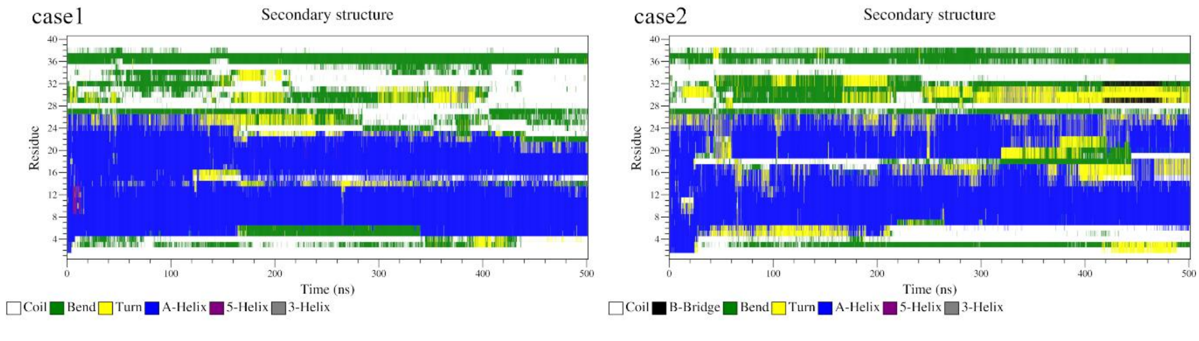


**Figure S5.** Time evolution of secondary structures of mesco-2 model1 in case1(left) and case2 (right) simulations with POPE:POPG membrane model, obtained by the DSSP [12] program.

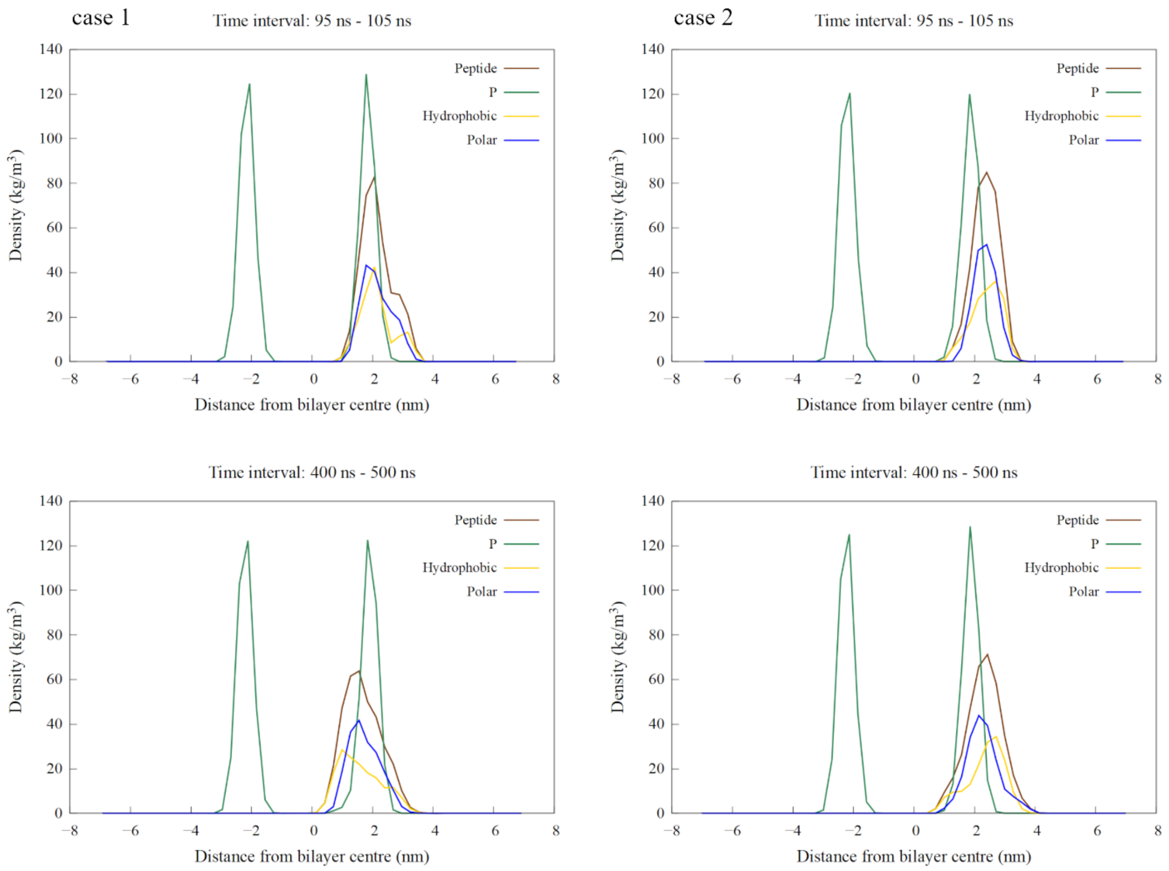


***Figure S6*** Density profiles calculated as an average over the time intervals: 90 ns –110 ns time interval (top), and 400 ns -500 ns time interval (bottom), for case1 on the left and case2 simulation on the right of a single peptide near the POPE:POPG membrane for *model1*. The profiles show the density of the entire peptide in brown, hydrophobic residues in yellow, polar residues in blue, and phosphorus atoms, representing the membrane's polar region, in green. The density profiles are calculated using the Gromacs [11] tool density,

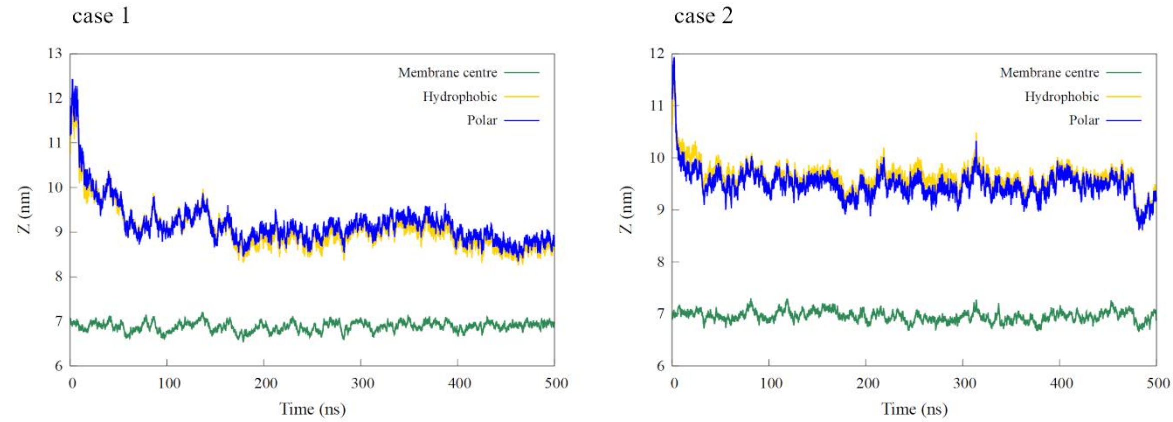


***Figure S7*** Centre of mass z coordinates of hydrophobic (yellow) and polar residues (blue) and P atoms representing the membrane (green), during case1 (left) and case2 (right) simulations of mesco-2 *model1* with the POPE:POPG membrane model calculated using Gromacs [11] tool *traj*.

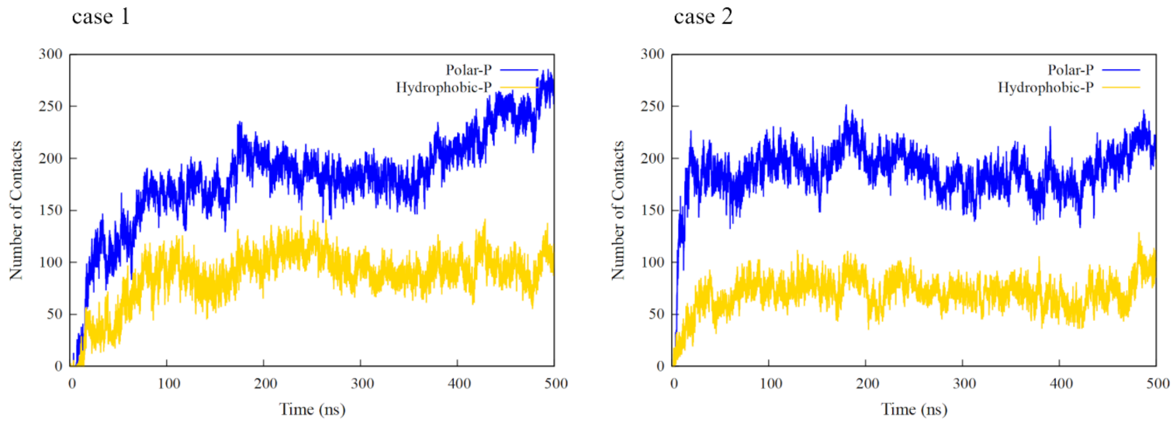


***Figure S8*** Number of contacts between P atoms and polar (blue) or hydrophobic (yellow) residues during case1 (left) and case2 (right) simulations of mesco-2 *model1* with the POPE:POPG membrane model calculated using Gromacs [11] tool *mindist*.

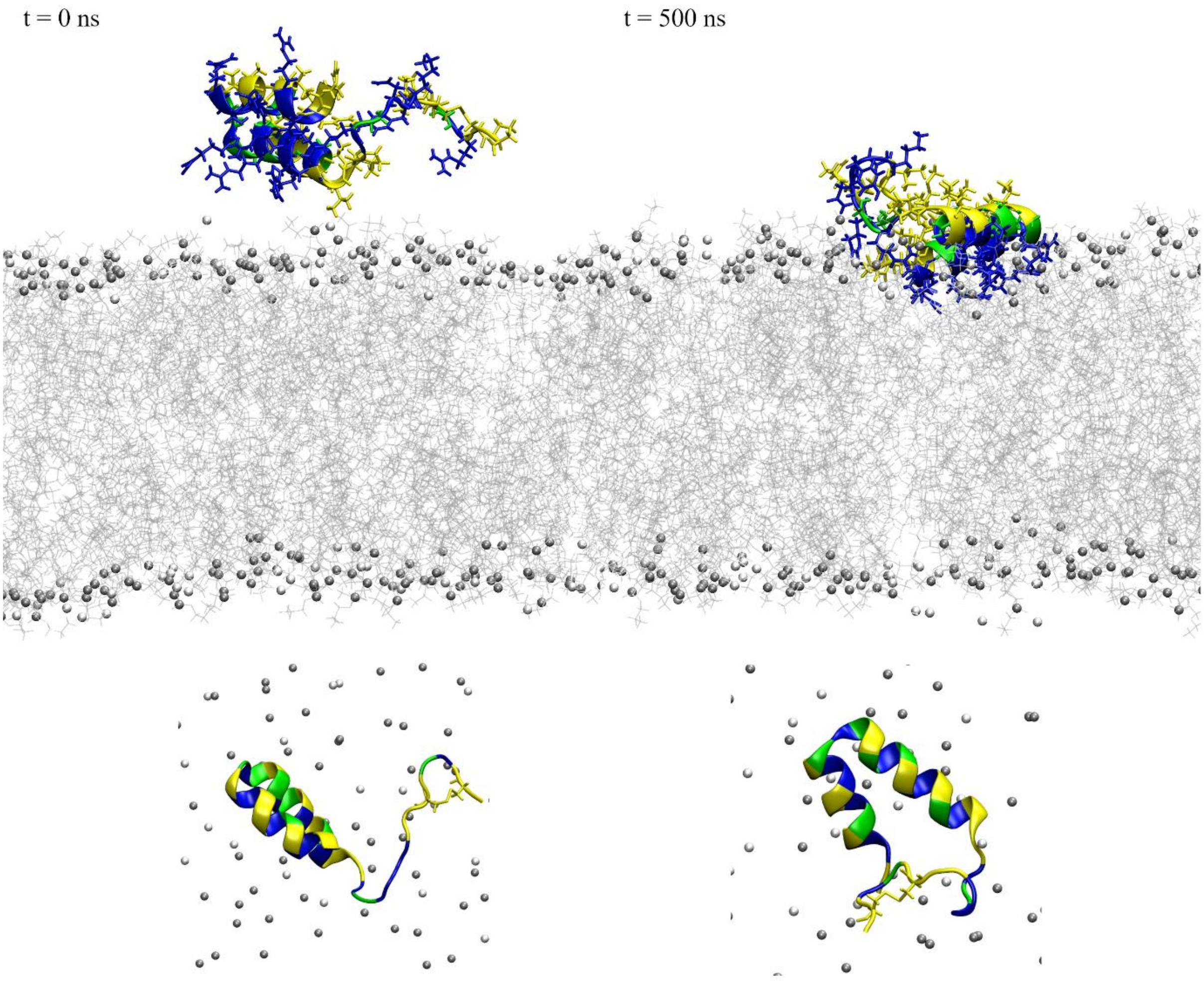


***Figure S9a*** Snapshots from the case1 simulation of a single peptide near POPE:POPG membrane for *model2,* with the initial conformation depicted on the left and the one at 500 ns simulation time on the right. The top row represents the side and bottom rows top view. The peptides are shown in a ribbon representation, with polar residues colored in blue, hydrophobic residues in yellow, and glycine in green. Lipids are represented in grey, with phosphorus atoms depicted as beads and acyl chains as lines. Water molecules and ions are excluded for clarity.

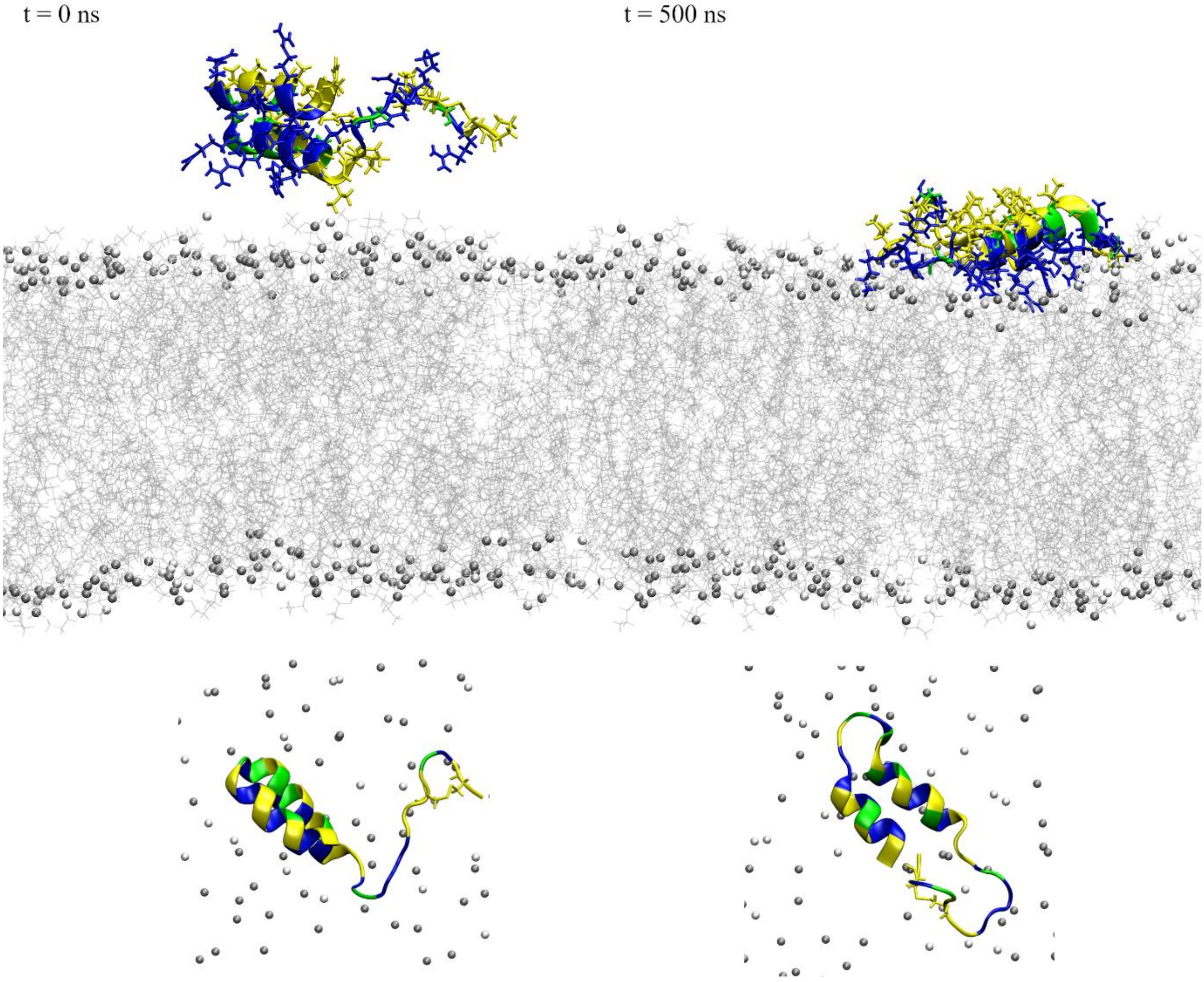


***Figure S9b*** Snapshots from the case2 simulation of a single peptide near POPE:POPG membrane for *model2,* with the initial conformation depicted on the left and the one at 500 ns simulation time on the right. The top row represents the side and bottom rows top view. The peptides are shown in a ribbon representation, with polar residues colored in blue, hydrophobic residues in yellow, and glycine in green. Lipids are represented in grey, with phosphorus atoms depicted as beads and acyl chains as lines. Water molecules and ions are excluded for clarity.

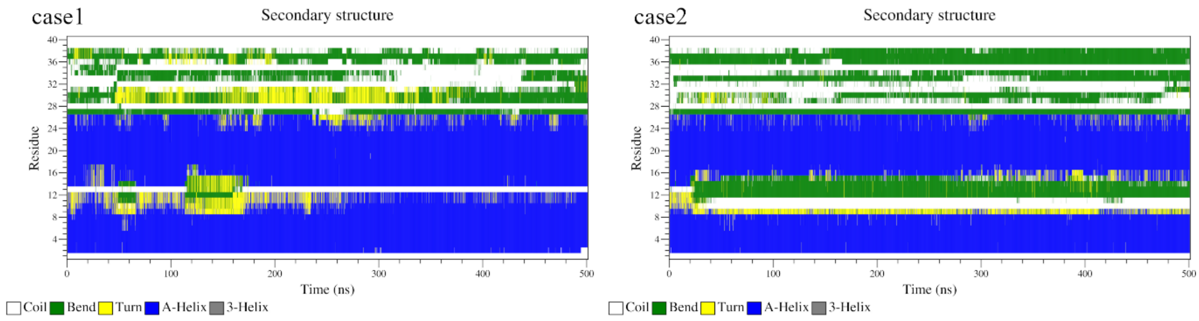


**Figure S10** Time evolution of secondary structures of mesco-2 model2 in case1(left) and case2 (right) simulations with POPE:POPG membrane model, obtained by the DSSP [12] program.

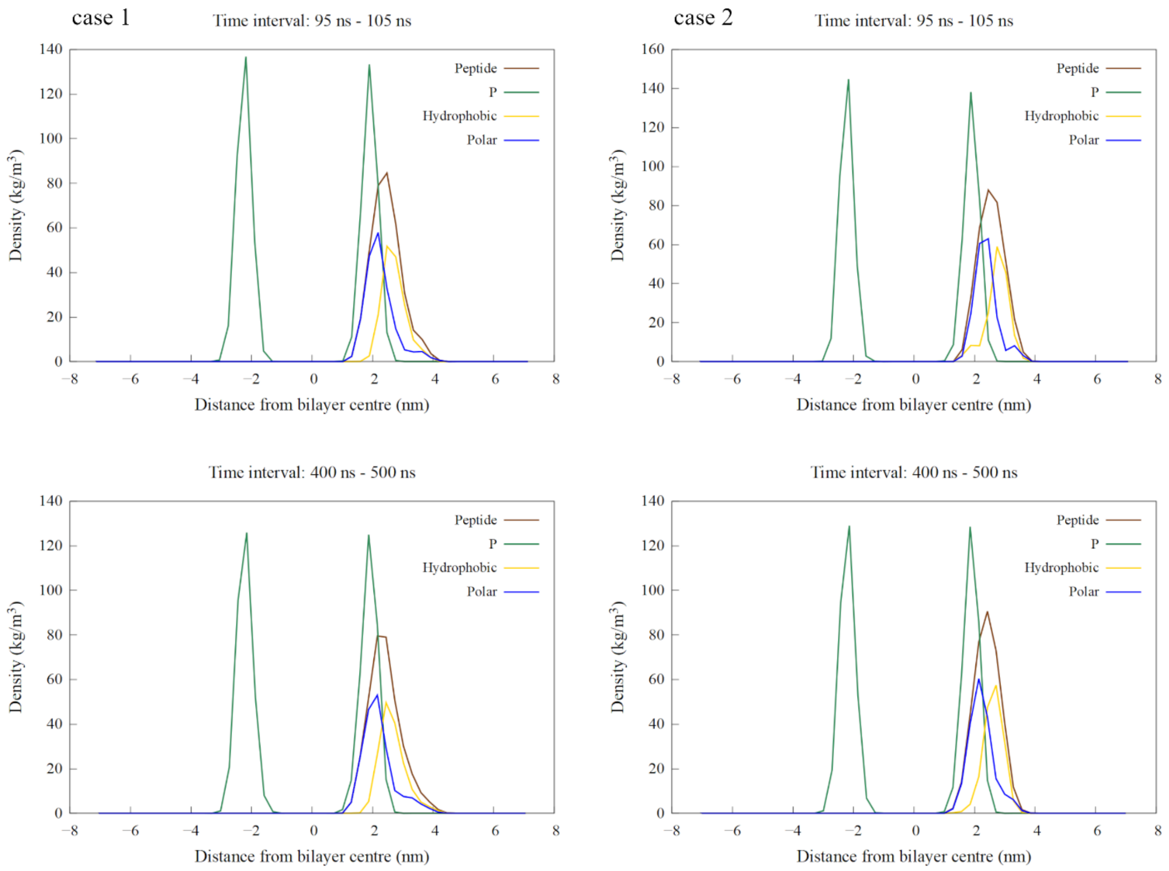


***Figure S11*** Density profiles calculated as an average over the time intervals: 90 ns –110 ns time interval (top), and 400 ns -500 ns time interval (bottom), for case1 on the left and case2 simulation on the right of a single peptide near the POPE:POPG membrane for *model1*. The profiles show the density of the entire peptide in brown, hydrophobic residues in yellow, polar residues in blue, and phosphorus atoms, representing the membrane's polar region, in green. The density profiles are calculated using the Gromacs [11] tool density,

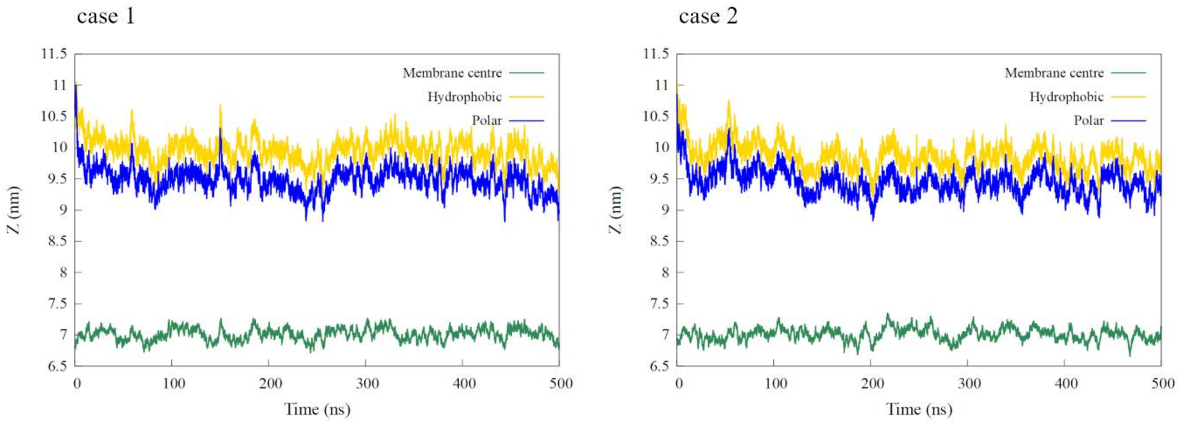


***Figure S12*** Centre of mass z coordinates of hydrophobic (yellow) and polar residues (blue) and P atoms representing the membrane (green), during case1 (left) and case2 (right) simulations of mesco-2 *model2* with the POPE:POPG membrane model calculated using Gromacs [11] tool *traj*.

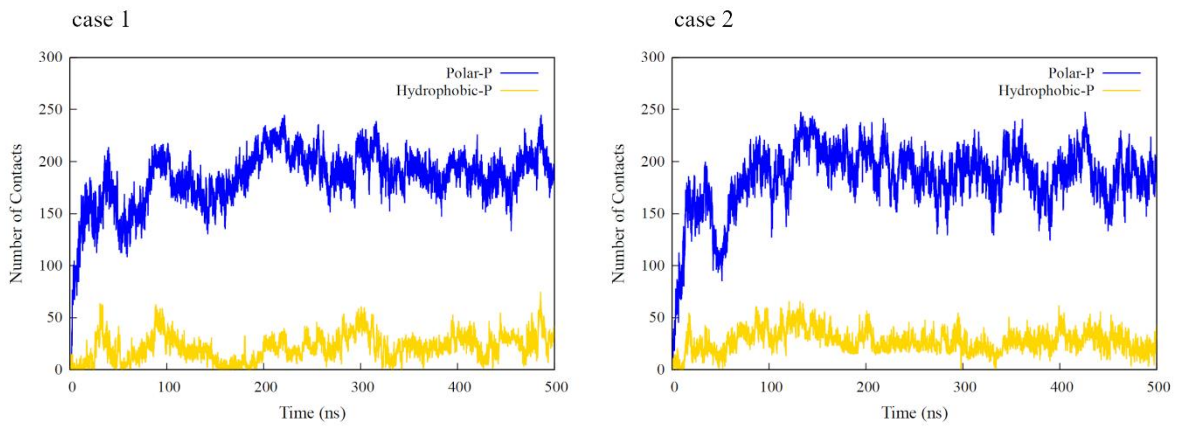


***Figure S13*** Number of contacts between P atoms and polar (blue) or hydrophobic (yellow) residues during case1 (left) and case2 (right) simulations of mesco-2 *model2* with the POPE:POPG membrane model calculated using Gromacs [11] tool *mindist.*

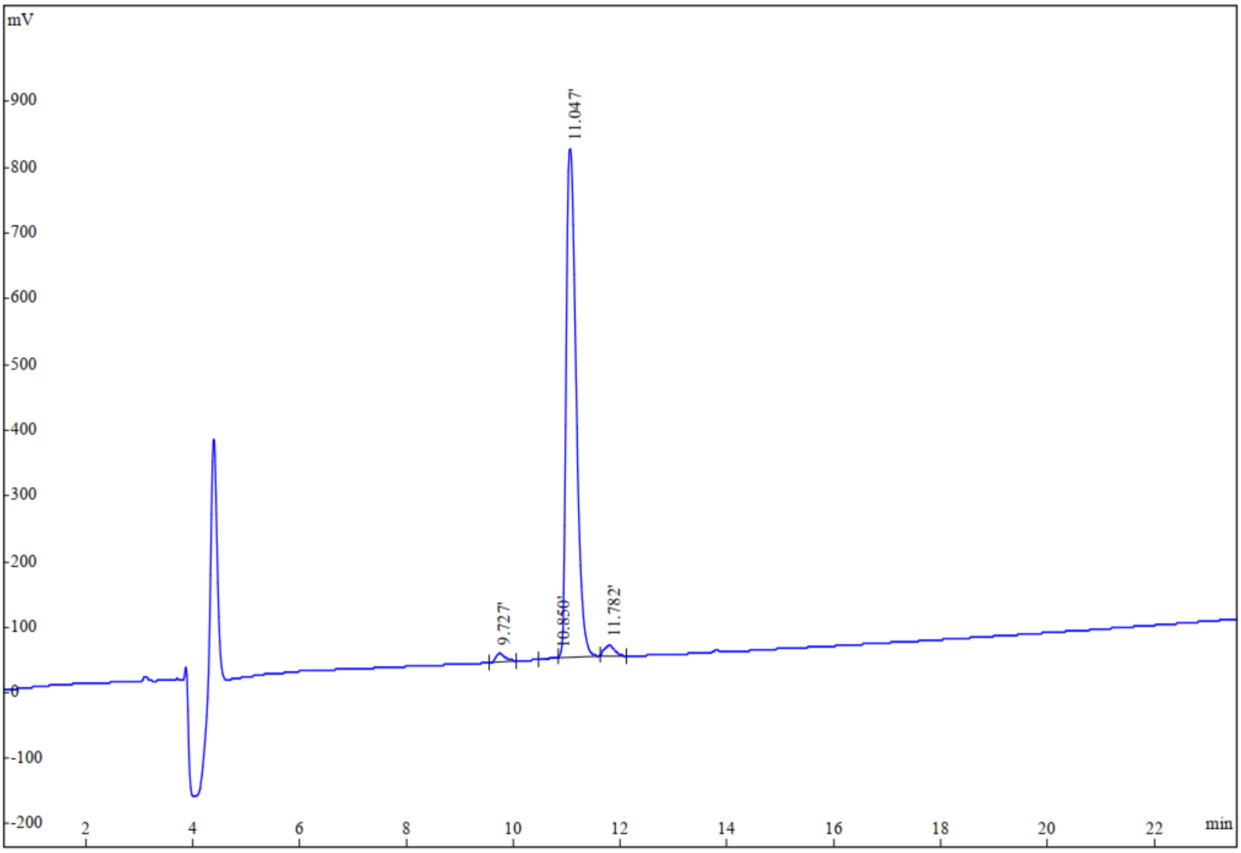


***Figure S14.*** Analytical RP-HPLC of mesco-2 peptide. Analytical column (5 µm, 4.6 × 250 mm) was used with 25–75% acetonitrile/0.1% TFA gradient in 25 min at 1 mL/min flow rate.

## References


[1] U. Gawde *et al.*, "CAMPR4: a database of natural and synthetic antimicrobial peptides," *Nucleic Acids Res,* vol. 51, no. D1, pp. D377-D383, Jan 6 2023, doi: 10.1093/nar/gkac933.

[2] N. Schaduangrat, C. Nantasenamat, V. Prachayasittikul, and W. Shoombuatong, "ACPred: A Computational Tool for the Prediction and Analysis of Anticancer Peptides," (in eng), *Molecules,* vol. 24, no. 10, May 22 2019, doi: 10.3390/molecules24101973.

[3] V. K. Sangaraju, N. T. Pham, L. Wei, X. Yu, and B. Manavalan, "mACPpred 2.0: Stacked Deep Learning for Anticancer Peptide Prediction with Integrated Spatial and Probabilistic Feature Representations," *Journal of Molecular Biology,* p. 168687, 2024/06/25/ 2024, doi: https://doi.org/10.1016/j.jmb.2024.168687.

[4] M. S. Khatun, M. M. Hasan, and H. Kurata, "PreAIP: Computational Prediction of Anti-inflammatory Peptides by Integrating Multiple Complementary Features," (in eng), *Front Genet,* vol. 10, p. 129, 2019, doi: 10.3389/fgene.2019.00129.

[5] P. B. Timmons and C. M. Hewage, "ENNAVIA is a novel method which employs neural networks for antiviral and anti-coronavirus activity prediction for therapeutic peptides," *Briefings in Bioinformatics,* vol. 22, no. 6, 2021, doi: 10.1093/bib/bbab258.

[6] N. Schaduangrat, C. Nantasenamat, V. Prachayasittikul, and W. Shoombuatong, "Meta-iAVP: A Sequence-Based Meta-Predictor for Improving the Prediction of Antiviral Peptides Using Effective Feature Representation," (in eng), *Int J Mol Sci,* vol. 20, no. 22, Nov 15 2019, doi: 10.3390/ijms20225743.

[7] P. K. Meher, T. K. Sahu, V. Saini, and A. R. Rao, "Predicting antimicrobial peptides with improved accuracy by incorporating the compositional, physico-chemical and structural features into Chou's general PseAAC," (in eng), *Sci Rep,* vol. 7, p. 42362, Feb 13 2017, doi: 10.1038/srep42362.

[8] B. Manavalan and M. C. Patra, "MLCPP 2.0: An Updated Cell-penetrating Peptides and Their Uptake Efficiency Predictor," *Journal of Molecular Biology,* vol. 434, no. 11, p. 167604, 2022/06/15/ 2022, doi: https://doi.org/10.1016/j.jmb.2022.167604.

[9] S. Gupta, A. K. Sharma, V. Shastri, M. K. Madhu, and V. K. Sharma, "Prediction of anti-inflammatory proteins/peptides: an insilico approach," (in eng), *J Transl Med,* vol. 15, no. 1, p. 7, Jan 6 2017, doi: 10.1186/s12967-016-1103-6.

[10] S. Gupta, P. Kapoor, K. Chaudhary, A. Gautam, R. Kumar, and G. P. Raghava, "Peptide toxicity prediction," (in eng), *Methods Mol Biol,* vol. 1268, pp. 143-57, 2015, doi: 10.1007/978-1-4939-2285-7_7.

[11] M. Abraham *et al.*, "GROMACS: High performance molecular simulations through multi-level parallelism from laptops to supercomputers," *SoftwareX,* vol. 1, 07/01 2015, doi: 10.1016/j.softx.2015.06.001.

[12] W. Kabsch and C. Sander, "Dictionary of protein secondary structure: Pattern recognition of hydrogen-bonded and geometrical features," *Biopolymers,* vol. 22, no. 12, pp. 2577-2637, 1983, doi: https://doi.org/10.1002/bip.360221211.
